# Genome Editing in Trees: From Multiple Repair Pathways to Long-Term Stability

**DOI:** 10.3389/fpls.2018.01732

**Published:** 2018-11-23

**Authors:** William Patrick Bewg, Dong Ci, Chung-Jui Tsai

**Affiliations:** ^1^Warnell School of Forestry and Natural Resources, Department of Genetics, and Department of Plant Biology, University of Georgia, Athens, GA, United States; ^2^Department of Bioscience and Biotechnology, Beijing Forestry University, Beijing, China

**Keywords:** mutagenesis, genome engineering, allele dose effect, *Populus*, biallelic, monoallelic, knockout

## Abstract

The CRISPR technology continues to diversify with a broadening array of applications that touch all kingdoms of life. The simplicity, versatility and species-independent nature of the CRISPR system offers researchers a previously unattainable level of precision and control over genomic modifications. Successful applications in forest, fruit and nut trees have demonstrated the efficacy of CRISPR technology at generating null mutations in the first generation. This eliminates the lengthy process of multigenerational crosses to obtain homozygous knockouts (KO). The high degree of genome heterozygosity in outcrossing trees is both a challenge and an opportunity for genome editing: a challenge because sequence polymorphisms at the target site can render CRISPR editing ineffective; yet an opportunity because the power and specificity of CRISPR can be harnessed for allele-specific editing. Examination of CRISPR/Cas9-induced mutational profiles from published tree studies reveals the potential involvement of multiple DNA repair pathways, suggesting that the influence of sequence context at or near the target sites can define mutagenesis outcomes. For commercial production of elite trees that rely on vegetative propagation, available data suggest an excellent outlook for stable CRISPR-induced mutations and associated phenotypes over multiple clonal generations.

## Introduction

CRISPR (Clustered Regularly Interspaced Short Palindromic Repeats)-based genome editing is rapidly becoming the system of choice for targeted mutagenesis in a growing variety of woody species, including forest trees. Forest trees are an invaluable commodity, providing fiber, energy, materials and climate buffering to the global community, and CRISPR has the potential to further enhance these important traits. Previous-generation methods for gene silencing in plants rely on expression of antisense RNAs, small interfering RNAs or microRNAs to base-pair with target mRNAs for degradation, often with unpredictable and unstable outcomes (Alessandra and Shihshieh, 2010). The specificity and efficiency of CRISPR for targeted DNA mutations, and the ease of adoption in virtually any species are behind the current revolution in genomic editing (Jiang and Doudna, 2017). Meanwhile, CRISPR’s popularity is driving the discovery and characterization of new CRISPR-associated (Cas) endonucleases with novel properties that make the system even more versatile (Burstein et al., 2016; Murovec et al., 2017). This review will focus on recent applications of CRISPR in woody species, with a special focus on forest trees, the mutation patterns observed at target sites, and the long-term stability of CRISPR/Cas9-edited outcomes.

## Crispr Applications in Woody Species

Phytoene desaturase (PDS) has been a popular marker for evaluating CRISPR in new study systems (Table 1). Its mutation disrupts chlorophyll biosynthesis, allowing for visual assessment of knockout (KO) efficiency. CRISPR/Cas9-induced albino mutants have been reported in poplar (Fan et al., 2015), citrus (Jia and Wang, 2014; Zhang et al., 2017), apple (Nishitani et al., 2016), grape (Nakajima et al., 2017), cassava (Odipio et al., 2017), coffee (Breitler et al., 2018), and kiwifruit (Wang Z. et al., 2018). Successful implementation of CRISPR has also been demonstrated by targeting potential developmental and biosynthesis pathway genes in grape (Ren et al., 2016) and the tropical tree *Parasponia andersonii* (van Zeijl et al., 2018; Table 1). New CRISPR reagents have been developed to expand genome editing capabilities. One such reagent, SaCas9 from *Staphylococcus aureus*, was shown to effectively generate mutations in Duncan grapefruit (Jia et al., 2017a). Compared to the most commonly used SpCas9 from *Streptococcus pyogenes*, SaCas9 is considerably smaller and recognizes a distinct 5′-NNGRRT protospacer adjacent motif (PAM) sequence (versus 5′-NGG of SpCas9). Using alternative CRISPR/Cas systems such as SaCas9 can increase the number of potential guide-RNA (gRNA) target sites, especially in AT-rich regions which may facilitate promoter editing.

**Table 1:** Summary of published CRISPR/Cas9-mediated knockout in woody species.

Species	Genes targeted	Mutation efficiencies	Transformation source tissue(s)	References
*Actinidia* chinensis (kiwifruit)	*PDS*	65–92%	*In vitro* leaves	Wang Z. et al., 2018^∗^
*Citrus sinensis, Citrus paradise, Poncirus trifoliate* x *Citrus sinensis* (citrus)	*Cs2g12470*, *Cs7g03360*, *LOB1* (promoter and gene) and *PDS*	3–100%	Greenhouse leaves^+^, *in vitro* epicotyl segments	Jia and Wang, 2014^∗+^; Jia et al., 2016, 2017a,b^∗+^; Peng et al., 2017^∗^; Zhang et al., 2017^∗^
*Coffea canephora* (coffee)	*PDS*	Up to 30%	Embryogenic calli	Breitler et al., 2018
*Malus domestica, Malus prunifolia* x *Malus pumila* (apple)	*DIPM1*, *DIPM2*, *DIPM4*, and *PDS*	Up to 32%	*In vitro* leaves, protoplastsˆ	Malnoy et al., 2016ˆ; Nishitani et al., 2016
*Manihot esculenta* (cassava)	*PDS*	97–99%	Embryogenic calli	Odipio et al., 2017^∗^
*Parasponia andersonii* (tropical tree)	*EIN2*, *HK4*, *NSP1* and *NSP2*	48–89%	Greenhouse tissues	van Zeijl et al., 2018
*Populus tomentosa*, *Populus tremula x alba*, *Populus tremula x tremuloides* (poplar)	*4CL1*, *4CL2*, *4CL5*, *AG1*, *AG2*, *BRC1-1*, *BRC2-1*, *DWF4*, *LFY*, *MYB57*, *MYB115*, *MYB156*, *MYB170*, *PDS*, and *WRKY18*	Up to 100%	*In vitro* leaves, *in vitro* shoots (leaf, stem and petiole pieces)	Fan et al., 2015; Zhou et al., 2015^∗^; Jiang et al., 2017; Wan et al., 2017; Wang et al., 2017; Xu et al., 2017^∗^; Yang et al., 2017; Elorriaga et al., 2018^∗^; Muhr et al., 2018^∗^; Shen et al., 2018^∗^
*Theobroma cacao* (cacao)	*NPR3*	Up to 27%	*In vitro* somatic embryo cotyledons	Fister et al., 2018^∗^
*Vitis vinifera* (grape)	*ldnDH*, *MLO-7*, *PDS,* and *WRKY52*	0.1–100%	Embryogenic calli, protoplastsˆ	Malnoy et al., 2016ˆ; Ren et al., 2016^∗^; Nakajima et al., 2017^∗^; Wang X. et al., 2018^∗^


*^∗^Indicates off-target mutations were assessed. All transformations performed via Agrobacterium unless otherwise stated. ^+^Xcc (Xanthomonas citri subsp. citri)-facilitated agroinfiltration. ^∧^Direct delivery of Cas9-gRNA ribonucleoproteins.*

Besides proof-of-concept studies, the CRISPR/Cas9 system has been used to develop disease resistant fruit trees with promising results (Table 1). The devastating citrus canker disease is caused by *Xanthomonas citri* subsp. *citri* (Xcc) through effector-activation of a canker susceptibility gene *LOB1* of the Lateral Organ Boundaries transcription factor family (Hu et al., 2014). When the *LOB1* promoter was targeted by CRISPR/Cas9 to disrupt the effector-binding element, canker symptoms after Xcc infection were reduced in Duncan grapefruit (Jia et al., 2016) and Wanjincheng orange (Peng et al., 2017). CRISPR-KO of *LOB1* also increases Xcc resistance in Duncan grapefruit (Jia et al., 2017b). KO-mutations in other susceptibility genes for powdery mildew and fire blight disease have also been achieved in grape and apple protoplasts, respectively (Malnoy et al., 2016), potentially allowing for the regeneration of disease-resistant plants. Several WRKY transcription factors involved in defense regulation have also been targeted for mutagenesis. CRISPR-KO of two positive regulators *PtrWRKY18* and *PtrWRKY35* compromised resistance to *Melampsora* rust in *Populus* (Jiang et al., 2017), whereas KO of grape *VvWRKY52* increased resistance to necrotrophic *Botrytis cinerea* (Wang X. et al., 2018).

To date, the greatest progress in woody species has been made with poplar, the first stably transformed tree to be genome-edited by CRISPR with high efficiency (Zhou et al., 2015). Allele-sensitive bioinformatics resources to facilitate genome editing in heterozygous species quickly followed, again based on the poplar system (Xue and Tsai, 2015; Xue et al., 2015). The majority of CRISPR studies in poplar have targeted phenylpropanoid metabolism or cell wall traits (Table 1). Mutations of individual 4-coumarate:CoA ligase (*4CL*) genes decreased the levels of structural (lignin) or non-structural (proanthocyanidin) phenylpropanoid polymers. CRISPR-KO of MYB transcription factors either increased (*PtoMYB156* and *PtrMYB57*) or decreased (*PtoMYB115* and *PtoMYB170*) phenylpropanoid flux, affecting in turn lignin deposition (*PtoMYB156* and *PtoMYB170*) or flavonoid accrual (*PtrMYB57* and *PtoMYB115*), respectively (Wan et al., 2017; Wang et al., 2017; Xu et al., 2017; Yang et al., 2017). Secondary cell wall synthesis was also compromised by CRISPR-KO of a brassinosteroid biosynthetic gene, supporting a role for brassinosteroids in wood formation (Shen et al., 2018). CRISPR-KO of *BRANCHED1-1* (*BRC1-1*) and *BRC1-2* belonging to the TCP family of transcription factors resulted in altered shoot architecture, and revealed an additional role of BRC2 in leaf development not previously reported for its *Arabidopsis* ortholog (Muhr et al., 2018). A recent study reported successful mutation of essential flowering genes in both male and female poplar genotypes (Elorriaga et al., 2018). The study also collated a large mutation dataset from over 500 transgenic events (Elorriaga et al., 2018) which should prove of value to understanding CRISPR/Cas editing patterns (see below). Although phenotypic evaluation of the flowering traits will require follow-on and multiyear studies in the field, the work underscores a powerful social application of CRISPR in containment of transgenic trees.

## Diverse Indel Profiles Indicative of cNHEJ, MMEJ, and TMEJ Activities

**FIGURE 1:**
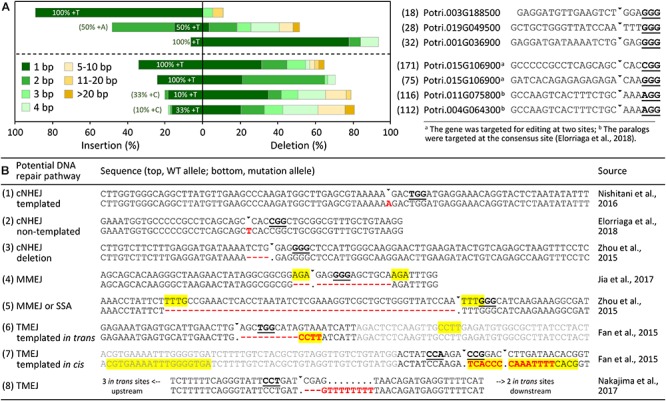
The mutation profiles and potential DNA repair pathways of CRISPR/Cas9-induced DSBs in *Populus* and other trees. **(A)** Distribution of mutation types at distinct genomic sites based on data from Zhou et al. (2015) (top panel) and Elorriaga et al. (2018) (bottom panel). The corresponding target genes and gRNA sequences are shown on the right, with allele number for each noted in parentheses. The percentages shown inside the +1 (1 bp insertion) bar indicate the fraction that were T insertions. The fraction of templated +1 insertions that deviate from T is shown in parentheses. **(B)** Representative examples of different mutation types and the potential DNA repair pathway involved in each case. PAM sequences are bold underlined, triangles denote predicted Cas9-cleavage sites, indels are shown in red, yellow-shaded regions denote microhomologies, and gray sequences in (6) and (7) were appended from *P. tomentosa* cDNA (GenBank accession KC954700) and *P. tremula* x *alba* 717 genomic sequences (Xue et al., 2015), respectively. Note, the region in (7) contains two overlapping target sites. For (8), there are five possible *in trans* template sites within introns of Phytozome (v12) gene model GSVIVG01016650001, the nearest one 640 bp upstream of the target site.

Small frameshift indels are the most common repair outcomes of single gRNA-directed Cas9 cleavage in trees, with 1 bp insertions (+1), especially +T and +A, predominant in many cases, similar to findings from other plants and animals (Bortesi et al., 2016). However, considerable variations and case-dependent repair outcomes are also noted, suggesting potential influences of target site sequences and/or their genomic contexts (Jacobs et al., 2015; Xu et al., 2015). Meta-analysis of mutation patterns across published tree studies is necessary to gain further insight, but that is made difficult by different reporting formats (not all studies report multi-allele data), and by the use of detection methods that differ in their sensitivity, accuracy, and allele discrimination (Sentmanat et al., 2018). We combined amplicon sequencing data from CRISPR-edited *P. tremula* x *alba* IRNA 717-1B4 (717) generated in our lab (Zhou et al., 2015) with the large 717 dataset from Elorriaga et al. (2018), along with manual inspection of other published tree studies for mutation profile analysis (Figure 1). In aggregate, +1 insertions constituted the greatest fraction of mutation types, followed by -1, and then -2, although stereotyped repair patterns are evident (Figure 1A). Interestingly, insertions were limited to +1 and +2 across all sites, whereas deletions spanned a much broader size range, though with decreasing frequencies for larger deletions.

Small mutagenic indels have often been ascribed to the classical non-homologous end-joining (cNHEJ) DNA repair pathway, but recent studies have demonstrated involvement of the alternative end-joining (alt-EJ) pathway as well (Rodgers and McVey, 2016). It now appears that cNHEJ contributes to the most common +1 insertions and other small indels, whereas larger indels are due to alt-EJ. This is based on studies where impaired cNHEJ drastically changed the repair outcomes of CRISPR/Cas9 in yeast, human cells and *Arabidopsis*, such that the typically predominant +1 insertions as well as other small (<3 bp) indels were greatly reduced, while rates of large indels increased, apparently independently of cNHEJ (van Overbeek et al., 2016; Shen et al., 2017; Lemos et al., 2018). In yeast, the vast majority of the +1 insertions from cNHEJ were templated from 1 bp 5′overhangs at the Cas9 cleavage site (fourth base from the PAM), and dependent on POL4, a low-fidelity X-family DNA polymerase with terminal transferase activity (Lemos et al., 2018). POL4-deficient yeast also lost +2 and +3 insertions, many of which are homonucleotides and apparently templated from the Cas9 cleavage site as well (Lemos et al., 2018). Templated insertions could also explain the majority of +1 events in a large Cas9-induced indel dataset from human cells (van Overbeek et al., 2016), suggesting a conserved mechanism underlying +1 insertions in CRISPR/Cas9-edited organisms (Lemos et al., 2018). In the combined 717 dataset, the majority of +1 insertions were +T as reported in many CRISPR studies. However, evidence in support of templated +1 insertions was weak, and appeared to be target site-dependent (Figure 1A). Clearly, much more data with greater target site diversity and coverage are necessary before a conclusion can been drawn, but such data from trees will require significant and perhaps community-wide efforts. Regardless, the small target site collection used in our analysis supports involvement of more than one mechanism for the commonly observed +T insertions, at least in *Populus*.

cNHEJ-independent repair likely involves different alt-EJ pathways, including microhomology-mediated end-joining (MMEJ), single-strand annealing (SSA), or polymerase theta (POLQ)-mediated end-joining (TMEJ) (Rodgers and McVey, 2016). Both MMEJ and SSA require end resection or unwinding to expose short homologous sequences for annealing (up to ∼20–30 bp for MMEJ and longer for SSA) and subsequent repair, and always result in deletions (Sfeir and Symington, 2015). The presence of microhomologous sequences at the deletion junctions can therefore serve as evidence of MMEJ/SSA repair. Indeed, microhomologies of 1–5 bp are readily identifiable in most of the deletion (≥5 bp) alleles we examined, but are rarely found for small deletions (3–4 bp) that might have arisen from cNHEJ (Figure 1B). MMEJ has also been associated with abnormal chromosomal translocations and inversions (Sfeir and Symington, 2015). Such modifications have been reported in several studies – including two from *Populus* – where two or more gRNAs were designed to target the same gene to produce large deletions (Fan et al., 2015; Elorriaga et al., 2018). Moreover, large deletions are sometimes accompanied by small insertions (Fan et al., 2015; Nakajima et al., 2017), a pattern that is characteristic of the recently discovered TMEJ pathway (Koole et al., 2014). TMEJ depends on POLQ, an error-prone A-family DNA polymerase that can extend microhomologies in a template-dependent (either *in cis* or *in trans*) or independent manner (Kent et al., 2016). TMEJ is the essential repair pathway in animal germ cells, as embryos of zebrafish *polq* mutants are hypersensitive to DSB-inducing treatments, with low levels of repair producing only +1 insertions (Thyme and Schier, 2016). In *Arabidopsis*, TMEJ is required for T-DNA integration following *Agrobacterium* transformation of either flowers or roots (van Kregten et al., 2016). We found evidence of *in cis* or *in trans* templated insertions in the complex indels reported for poplar and grape (Figure 1B; Fan et al., 2015; Nakajima et al., 2017), supporting an active TMEJ pathway in somatic cells of plants.

Examination of published mutation profiles of *Populus* and other tree species suggests differential involvement of multiple repair pathways, probably with cNHEJ contributing to +1, +2 and small (1–4 bp) deletions, MMEJ (and SSA) to larger deletions, and TMEJ to complex indels (Figure 1B). The varying dependency of these pathways on sequence contexts (microhomologies) likely underpins the non-random nature of CRISPR/Cas9 repair outcomes reported in many studies, including trees (Jacobs et al., 2015; van Overbeek et al., 2016; Vu et al., 2017; Elorriaga et al., 2018). Incorporation of microhomology modeling into the gRNA design workflow (Bae et al., 2014; Segar et al., 2015) should enable prediction of potential DNA repair outcomes for informed selection of target sites.

## Long-Term Stability of Crispr-Edited Trees Through Vegetative Propagation

For many herbaceous species where CRISPR editing efficiencies are low, or where monoallelic/mosaic mutations predominate in the first-generation (T0) transformants, multi-generation progeny screening is necessary to obtain homozygous mutants (Xu et al., 2015). Although initial transmission rates vary depending on the study system and the nature of CRISPR-induced (somatic or germinal) mutations carried by the founder plant, stable mutation inheritance can be expected once homozygous lines are obtained, as reported for *Arabidopsis*, rice, tomato and potato (Brooks et al., 2014; Feng et al., 2014; Zhou et al., 2014; Butler et al., 2015). For woody perennials, however, the issues are rather different. Cross-generational screening is difficult to implement for transgenic trees owing to their long generation times and strict regulation of flowering transgenic trees (Strauss et al., 2015). The predominantly outcrossing nature of trees, many of which are also dioecious, adds further challenge to rapid-cycle breeding and introgression of CRISPR-derived mutations into elite germplasms. While advances of early-flowering induction in contained environments (Hoenicka et al., 2014; Klocko et al., 2016) promise to accelerate progress, commercial production of many forest, fruit and nut trees relies on clonal propagation of elite genotypes. For woody perennials, therefore, it is pertinent to address long-term stability of CRISPR editing, both on-target and off-target, in vegetatively-propagated T0 transformants.

In theory, CRISPR-induced DNA modifications should lead to permanent mutations in edited cells that can be inherited mitotically during clonal propagation, yet experimental data are rare. One study used tissue culture to clone CRISPR-derived mutations from T0 diploid and tetraploid potato, and reported stable maintenance of targeted mutations across clonal generations, and in three selected cases, through the germline as well (Butler et al., 2015). In that study, however, somatic mutations were prevalent in T0 plants, as fewer than half of the originally-detected mutation types were captured as single mutations in clonally-propagated plants (Butler et al., 2015). The high levels of somatic mutations likely reflect a high proportion of chimeras, a common problem in tissue culture when plants are regenerated from multiple cells, in this case, with heterogenomic modifications. Fortunately for *Populus*, the proven efficiency of CRISPR (Zhou et al., 2015; Elorriaga et al., 2018) means null mutations with biallelic KO can be readily obtained in T0 transformants and stably inherited through clonal propagation. Wang et al. (2017) reported faithful maintenance of *PtoMYB115* mutations in tissue culture-propagated *Populus tomentosa* somaclones, though in one case low frequencies of new mutations not seen in the parent line were detected, indicative of chimeras. Similarly, the CRISPR editing outcomes of *BRC1-1* and *BRC2-1* were also stable over multiple cycles of vegetative propagation in tissue culture (Muhr et al., 2018). We have maintained a subset of the previously-characterized 717 mutants (Zhou et al., 2015) in the greenhouse for over 4 years by repeatedly cutting back the original transformants and/or propagation using rooted cuttings. The reddish-brown wood discoloration of lignin-reduced *4cl1* mutants has been stable in all re-sprouted shoots or clonally-propagated plants. Repeated amplicon-sequencing of randomly selected lines re-confirmed the targeted *4CL1* mutations 4 years later, with no off-target changes to the paralogous *4CL5* (Supplementary Table S1). Another group of transgenic plants harbors a non-functional gRNA for *4CL5* due to SNPs (one per allele) between the genome-sequenced *P. trichocarpa* and the transformation host 717 that prevented Cas9 cleavage as confirmed by amplicon sequencing (Zhou et al., 2015). It should be noted that one of the 717 SNPs alters the PAM site from NGG to NGA, the latter is a non-canonical PAM of SpCas9 thought to cause off-target cleavage in human cells (Zhang Y. et al., 2014b). Reanalyzing this group of plants will inform as to whether the imperfectly-matched *4CL5*-gRNA exhibited any off-target activity over the long term. We found no evidence of CRISPR/Cas9 cleavage after 4 years of coppicing and regrowth (Supplementary Table S1). These findings echo other tree studies that showed no or very rare off-targeting (Jia et al., 2017a; Nakajima et al., 2017; Elorriaga et al., 2018; see also Table 1), as well as reports from *Arabidopsis*, rice and tomato based on whole-genome re-sequencing (Feng et al., 2014; Zhang et al., 2014a; Peterson et al., 2016; Rodríguez-Leal et al., 2017). The data provide support for long-term stability and specificity of CRISPR/Cas9-mediated mutagenesis, with extremely low off-target potential during vegetative propagation in poplar.

## Broad-Spectrum Mutagenesis Beyond KO

Nullizygous mutations harboring either identical (homozygous) or distinct (heterozygous) mutations in all alleles of the genome are the ideal repair outcomes for gene KO investigation. However, monoallelic, in-frame and/or mosaic mutations can expand the phenotypic spectrum to enhance the power of functional analysis. For instance, transgenic grapevine with monoallelic mutations of a defense-related *WRKY* gene exhibited intermediate levels of disease resistance between WT and biallelic mutants (Wang X. et al., 2018). Similarly, monoallelic or in-frame mutations of PDS led to partial albino phenotypes in both poplar and apple (Fan et al., 2015; Nishitani et al., 2016). Given the abundance of duplicate genes in plant genomes, and the proven successes of CRISPR in multi-allele as well as allele-specific editing (Jia et al., 2016; Elorriaga et al., 2018), there is exciting potential to exploit CRISPR for development of allelic series mutations to address functional redundancy of duplicate genes or tandem repeats, and to investigate the allele-dose response of agronomic traits. Thus, the ability to generate novel germplasms is invaluable not only for tree improvement but also for basic functional genomics research.

In contrast to CRISPR-mediated KO, site-specific gene targeting or replacement remains a major challenge in plants, due to the inefficient homology-directed repair pathway. Geminivirus replicons have been shown to increase site-specific gene knockin (KI) efficiencies by orders of magnitude in tobacco, tomato and hexaploid wheat (Baltes et al., 2014; Čermák et al., 2015; Gil-Humanes et al., 2017). In animal systems, the MMEJ and SSA pathways, along with a new CRISPR/Cpf1 system have been harnessed for targeted KI with success (Sakuma et al., 2016; Tóth et al., 2016). These and other emerging approaches represent promising options for developing efficient KI systems in trees. Finally, many economically important tree species or genotypes remain recalcitrant to transformation and/or tissue culture regeneration, hindering applications of CRISPR. Recent breakthroughs in morphogenic regulator-mediated regeneration (Lowe et al., 2016, 2018) have already stimulated similar research in trees. Direct delivery of pre-assembled Cas9-gRNA ribonucleoproteins into protoplasts for genome editing as already deployed in apple and grape offers a transgene-free alternative to *Agrobacterium* transformation (Malnoy et al., 2016). At the present time, however, protoplast regeneration for other tree species remains a challenge. There is strong incentive to overcome this challenge since avoiding the footprint of foreign DNA and the associated negative perceptions will improve the outlook for integration of CRISPR technology with commercial deployment of designer trees.

## Author Contributions

C-JT conceived the idea. WPB, DC, and C-JT collected background information and analyzed data. WPB and C-JT wrote the manuscript with contributions from DC. All authors approved the manuscript.

## Conflict of Interest Statement

The authors declare that the research was conducted in the absence of any commercial or financial relationships that could be construed as a potential conflict of interest.

## References

[B1] Alessandra F. Shihshieh H. (2010). Tapping RNA silencing pathways for plant biotechnology. *Plant Biotechnol. J.* 8 655–677. 10.1111/j.1467-7652.2010.00505.x 20331529

[B2] Bae S. Kweon J. Kim H. S. Kim J.-S. (2014). Microhomology-based choice of Cas9 nuclease target sites. *Nat. Methods* 11:705. 10.1038/nmeth.3015 24972169

[B3] Baltes N. J. Gil-Humanes J. Cermak T. Atkins P. A. Voytas D. F. (2014). DNA replicons for plant genome engineering. *Plant Cell* 26 151–163. 10.1105/tpc.113.119792 24443519 PMC3963565

[B4] Bortesi L. Zhu C. Zischewski J. Perez L. Bassié L. Nadi R. (2016). Patterns of CRISPR/Cas9 activity in plants, animals and microbes. *Plant Biotechnol. J.* 14 2203–2216. 10.1111/pbi.12634 27614091 PMC5103219

[B5] Breitler J.-C. Dechamp E. Campa C. Zebral Rodrigues L. A. Guyot R. Marraccini P. (2018). CRISPR/Cas9-mediated efficient targeted mutagenesis has the potential to accelerate the domestication of *Coffea canephora*. *Plant Cell Tissue Organ Cult.* 134 383–394. 10.1007/s11240-018-1429-2

[B6] Brooks C. Nekrasov V. Lippman Z. B. Van Eck J. (2014). Efficient gene editing in tomato in the first generation using the clustered regularly interspaced short palindromic repeats/CRISPR-associated9 system. *Plant Physiol.* 166 1292–1297. 10.1104/pp.114.247577 25225186 PMC4226363

[B7] Burstein D. Harrington L. B. Strutt S. C. Probst A. J. Anantharaman K. Thomas B. C. (2016). New CRISPR–Cas systems from uncultivated microbes. *Nature* 542 237–241. 10.1038/nature21059 28005056 PMC5300952

[B8] Butler N. M. Atkins P. A. Voytas D. F. Douches D. S. (2015). Generation and inheritance of targeted mutations in potato (*Solanum tuberosum* L.) using the CRISPR/Cas system. *PLoS One* 10:e0144591. 10.1371/journal.pone.0144591 26657719 PMC4684367

[B9] Čermák T. Baltes N. J. Čegan R. Zhang Y. Voytas D. F. (2015). High-frequency, precise modification of the tomato genome. *Genome Biol.* 16:232. 10.1186/s13059-015-0796-9 26541286 PMC4635538

[B10] Elorriaga E. Klocko A. L. Ma C. Strauss S. H. (2018). Variation in mutation spectra among CRISPR/Cas9 mutagenized poplars. *Front. Plant Sci.* 9:594. 10.3389/fpls.2018.00594 29868058 PMC5949366

[B11] Fan D. Liu T. Li C. Jiao B. Li S. Hou Y. (2015). Efficient CRISPR/Cas9-mediated targeted mutagenesis in Populus in the first generation. *Sci. Rep.* 5:12217. 10.1038/srep12217 26193631 PMC4507398

[B12] Feng Z. Mao Y. Xu N. Zhang B. Wei P. Yang D.-L. (2014). Multigeneration analysis reveals the inheritance, specificity, and patterns of CRISPR/Cas-induced gene modifications in Arabidopsis. *Proc. Natl. Acad. Sci. U.S.A.* 111 4632–4637. 10.1073/pnas.1400822111 24550464 PMC3970504

[B13] Fister A. S. Landherr L. Maximova S. N. Guiltinan M. J. (2018). Transient expression of CRISPR/Cas9 machinery targeting TcNPR3 enhances defense response in *Theobroma cacao. Front. Plant Sci.* 9:268. 10.3389/fpls.2018.00268 29552023 PMC5841092

[B14] Gil-Humanes J. Wang Y. Liang Z. Shan Q. Ozuna C. V. Sánchez-León S. (2017). High-efficiency gene targeting in hexaploid wheat using DNA replicons and CRISPR/Cas9. *Plant J.* 89 1251–1262. 10.1111/tpj.13446 27943461 PMC8439346

[B15] Hoenicka H. Lehnhardt D. Nilsson O. Hanelt D. Fladung M. (2014). Successful crossings with early flowering transgenic poplar: interspecific crossings, but not transgenesis, promoted aberrant phenotypes in offspring. *Plant Biotechnol. J.* 12 1066–1074. 10.1111/pbi.12213 24975279

[B16] Hu Y. Zhang J. Jia H. Sosso D. Li T. Frommer W. B. (2014). Lateral organ boundaries 1 is a disease susceptibility gene for citrus bacterial canker disease. *Proc. Natl. Acad. Sci. U.S.A.* 111 E521–E529. 10.1073/pnas.1313271111 24474801 PMC3910620

[B17] Jacobs T. B. Lafayette P. R. Schmitz R. J. Parrott W. A. (2015). Targeted genome modifications in soybean with CRISPR/Cas9. *BMC Biotechnol.* 15:16. 10.1186/s12896-015-0131-2 25879861 PMC4365529

[B18] Jia H. Orbovic V. Jones J. B. Wang N. (2016). Modification of the PthA4 effector binding elements in Type I CsLOB1 promoter using Cas9/sgRNA to produce transgenic Duncan grapefruit alleviating XccΔpthA4:dCsLOB1.3 infection. *Plant Biotechnol. J.* 14 1291–1301. 10.1111/pbi.12495 27071672 PMC11389130

[B19] Jia H. Wang N. (2014). Targeted genome editing of sweet orange using Cas9/sgRNA. *PLoS One* 9:e93806. 10.1371/journal.pone.0093806 24710347 PMC3977896

[B20] Jia H. Xu J. Orbovic V. Zhang Y. Wang N. (2017a). Editing citrus genome via SaCas9/sgRNA system. *Front. Plant Sci.* 8:2135. 10.3389/fpls.2017.02135 29312390 PMC5732962

[B21] Jia H. Zhang Y. Orbović V. Xu J. White F. F. Jones J. B. (2017b). Genome editing of the disease susceptibility gene CsLOB1 in citrus confers resistance to citrus canker. *Plant Biotechnol. J.* 15 817–823. 10.1111/pbi.12677 27936512 PMC5466436

[B22] Jiang F. Doudna J. A. (2017). CRISPR–Cas9 structures and mechanisms. *Annu. Rev. Biophys.* 46 505–529. 10.1146/annurev-biophys-062215-010822 28375731

[B23] Jiang Y. Guo L. Ma X. Zhao X. Jiao B. Li C. (2017). The WRKY transcription factors PtrWRKY18 and PtrWRKY35 promote Melampsora resistance in Populus. *Tree Physiol.* 37 665–675. 10.1093/treephys/tpx008 28338710

[B24] Kent T. Mateos-Gomez P. A. Sfeir A. Pomerantz R. T. (2016). Polymerase 𝜃 is a robust terminal transferase that oscillates between three different mechanisms during end-joining. *eLife* 5:e13740. 10.7554/eLife.13740 27311885 PMC4912351

[B25] Klocko A. L. Ma C. Robertson S. Esfandiari E. Nilsson O. Strauss S. H. (2016). FT overexpression induces precocious flowering and normal reproductive development in Eucalyptus. *Plant Biotechnol. J.* 14 808–819. 10.1111/pbi.12431 26132805 PMC11389084

[B26] Koole W. Van Schendel R. Karambelas A. E. Van Heteren J. T. Okihara K. L. Tijsterman M. (2014). A Polymerase Theta-dependent repair pathway suppresses extensive genomic instability at endogenous G4 DNA sites. *Nat. Commun.* 5:3216. 10.1038/ncomms4216 24496117

[B27] Lemos B. R. Kaplan A. C. Bae J. E. Ferrazzoli A. E. Kuo J. Anand R. P. (2018). CRISPR/Cas9 cleavages in budding yeast reveal templated insertions and strand-specific insertion/deletion profiles. *Proc. Natl. Acad. Sci. U.S.A.* 115 E2040–E2047. 10.1073/pnas.1716855115 29440496 PMC5834694

[B28] Lowe K. La Rota M. Hoerster G. Hastings C. Wang N. Chamberlin M. (2018). Rapid genotype “independent” *Zea mays* L. (maize) transformation via direct somatic embryogenesis. *In Vitro Cell. Dev. Biol.* 54 240–252. 10.1007/s11627-018-9905-2 29780216 PMC5954046

[B29] Lowe K. Wu E. Wang N. Hoerster G. Hastings C. Cho M.-J. (2016). Morphogenic regulators Baby boom and Wuschel improve monocot transformation. *Plant Cell* 28 1998–2015. 10.1105/tpc.16.00124 27600536 PMC5059793

[B30] Malnoy M. Viola R. Jung M. H. Koo O. J. Kim S. Kim J. S. (2016). DNA-free genetically edited grapevine and apple protoplast using CRISPR/Cas9 ribonucleoproteins. *Front. Plant Sci.* 7:1904. 10.3389/fpls.2016.01904 28066464 PMC5170842

[B31] Muhr M. Paulat M. Awwanah M. Brinkkötter M. Teichmann T. (2018). CRISPR/Cas9-mediated knockout of Populus BRANCHED1 and BRANCHED2 orthologs reveals a major function in bud outgrowth control. *Tree Physiol.* 38 1588–1597. 10.1093/treephys/tpy088 30265349

[B32] Murovec J. Pirc Ž Yang B. (2017). New variants of CRISPR RNA-guided genome editing enzymes. *Plant Biotechnol. J.* 15 917–926. 10.1111/pbi.12736 28371222 PMC5506654

[B33] Nakajima I. Ban Y. Azuma A. Onoue N. Moriguchi T. Yamamoto T. (2017). CRISPR/Cas9-mediated targeted mutagenesis in grape. *PLoS One* 12:e0177966. 10.1371/journal.pone.0177966 28542349 PMC5436839

[B34] Nishitani C. Hirai N. Komori S. Wada M. Okada K. Osakabe K. (2016). Efficient genome editing in apple using a CRISPR/Cas9 system. *Sci. Rep.* 6:31481. 10.1038/srep31481 27530958 PMC4987624

[B35] Odipio J. Alicai T. Ingelbrecht I. Nusinow D. A. Bart R. Taylor N. J. (2017). Efficient CRISPR/Cas9 genome editing of phytoene desaturase in cassava. *Front. Plant Sci.* 8:1780. 10.3389/fpls.2017.01780 29093724 PMC5651273

[B36] Peng A. Chen S. Lei T. Xu L. He Y. Wu L. (2017). Engineering canker-resistant plants through CRISPR/Cas9-targeted editing of the susceptibility gene CsLOB1 promoter in citrus. *Plant Biotechnol. J.* 15 1509–1519. 10.1111/pbi.12733 28371200 PMC5698050

[B37] Peterson B. A. Haak D. C. Nishimura M. T. Teixeira P. J. James S. R. Dangl J. L. (2016). Genome-wide assessment of efficiency and specificity in CRISPR/Cas9 mediated multiple site targeting in Arabidopsis. *PLoS One* 11:e0162169. 10.1371/journal.pone.0162169 27622539 PMC5021288

[B38] Ren C. Liu X. Zhang Z. Wang Y. Duan W. Li S. (2016). CRISPR/Cas9-mediated efficient targeted mutagenesis in Chardonnay (*Vitis vinifera* L.). *Sci. Rep.* 6:32289. 10.1038/srep32289 27576893 PMC5006071

[B39] Rodgers K. McVey M. (2016). Error-prone repair of DNA double-strand breaks. *J. Cell. Physiol.* 231 15–24. 10.1002/jcp.25053 26033759 PMC4586358

[B40] Rodríguez-Leal D. Lemmon Z. H. Man J. Bartlett M. E. Lippman Z. B. (2017). Engineering quantitative trait variation for crop improvement by genome editing. *Cell* 171 470.e–480.e. 10.1016/j.cell.2017.08.030 28919077

[B41] Sakuma T. Nakade S. Sakane Y. Suzuki K.-I. T. Yamamoto T. (2016). MMEJ-assisted gene knock-in using TALENs and CRISPR-Cas9 with the PITCh systems. *Nat. Protoc.* 11 118–133. 10.1038/nprot.2015.140 26678082

[B42] Segar M. W. Sakofsky C. J. Malkova A. Liu Y. (2015). MMBIRFinder: a tool to detect microhomology-mediated break-induced replication. *IEEE/ACM Trans. Comput. Biol. Bioinform.* 12 799–806. 10.1109/TCBB.2014.2359450 26357319 PMC4857593

[B43] Sentmanat M. F. Peters S. T. Florian C. P. Connelly J. P. Pruett-Miller S. M. (2018). A survey of validation strategies for CRISPR-Cas9 editing. *Sci. Rep.* 8:888. 10.1038/s41598-018-19441-8 29343825 PMC5772360

[B44] Sfeir A. Symington L. S. (2015). Microhomology-mediated end joining: a back-up survival mechanism or dedicated pathway? *Trends Biochem. Sci.* 40 701–714. 10.1016/j.tibs.2015.08.006 26439531 PMC4638128

[B45] Shen H. Strunks G. D. Klemann B. J. P. M. Hooykaas P. J. J. De Pater S. (2017). CRISPR/Cas9-induced double-strand break repair in Arabidopsis nonhomologous end-joining mutants. *G3* 7 193–202. 10.1534/g3.116.035204 27866150 PMC5217109

[B46] Shen Y. Li Y. Xu D. Yang C. Li C. Luo K. (2018). Molecular cloning and characterization of a brassinosteriod biosynthesis-related gene PtoDWF4 from *Populus tomentosa*. *Tree Physiol.* 38 1424–1436. 10.1093/treephys/tpy027 29579304

[B47] Strauss S. H. Costanza A. Séguin A. (2015). Genetically engineered trees: paralysis from good intentions. *Science* 349 794–795. 10.1126/science.aab0493 26293942

[B48] Thyme S. B. Schier A. F. (2016). Polq-mediated end joining is essential for surviving DNA double-strand breaks during early zebrafish development. *Cell Rep.* 15 707–714. 10.1016/j.celrep.2016.03.072 27149851 PMC5063659

[B49] Tóth E. Weinhardt N. Bencsura P. Huszár K. Kulcsár P. I. Tálas A. (2016). Cpf1 nucleases demonstrate robust activity to induce DNA modification by exploiting homology directed repair pathways in mammalian cells. *Biol. Direct* 11:46. 10.1186/s13062-016-0147-0 27630115 PMC5024423

[B50] van Kregten M. De Pater S. Romeijn R. Van Schendel R. Hooykaas P. J. J. Tijsterman M. (2016). T-DNA integration in plants results from polymerase-𝜃-mediated DNA repair. *Nat. Plants* 2:16164. 10.1038/nplants.2016.164 27797358

[B51] van Overbeek M. Capurso D. Carter M. M. Thompson M. S. Frias E. Russ C. (2016). DNA repair profiling reveals nonrandom outcomes at Cas9-mediated breaks. *Mol. Cell.* 63 633–646. 10.1016/j.molcel.2016.06.037 27499295

[B52] van Zeijl A. Wardhani T. A. K. Seifi Kalhor M. Rutten L. Bu F. Hartog M. (2018). CRISPR/Cas9-mediated mutagenesis of four putative symbiosis genes of the tropical tree *Parasponia andersonii* reveals novel phenotypes. *Front. Plant Sci.* 9:284. 10.3389/fpls.2018.00284 29559988 PMC5845686

[B53] Vu G. T. H. Cao H. X. Fauser F. Reiss B. Puchta H. Schubert I. (2017). Endogenous sequence patterns predispose the repair modes of CRISPR/Cas9-induced DNA double-stranded breaks in *Arabidopsis thaliana.* *Plant J.* 92 57–67. 10.1111/tpj.13634 28696528

[B54] Wan S. Li C. Ma X. Luo K. (2017). PtrMYB57 contributes to the negative regulation of anthocyanin and proanthocyanidin biosynthesis in poplar. *Plant Cell Rep.* 36 1263–1276. 10.1007/s00299-017-2151-y 28523445

[B55] Wang L. Ran L. Hou Y. Tian Q. Li C. Liu R. (2017). The transcription factor MYB115 contributes to the regulation of proanthocyanidin biosynthesis and enhances fungal resistance in poplar. *New Phytol.* 215 351–367. 10.1111/nph.14569 28444797

[B56] Wang X. Tu M. Wang D. Liu J. Li Y. Li Z. (2018). CRISPR/Cas9-mediated efficient targeted mutagenesis in grape in the first generation. *Plant Biotechnol. J.* 16 844–855. 10.1111/pbi.12832 28905515 PMC5866948

[B57] Wang Z. Wang S. Li D. Zhang Q. Li L. Zhong C. (2018). Optimized paired-sgRNA/Cas9 cloning and expression cassette triggers high-efficiency multiplex genome editing in kiwifruit. *Plant Biotechnol. J.* 16 1424–1433. 10.1111/pbi.12884 29331077 PMC6041439

[B58] Xu C. Fu X. Liu R. Guo L. Ran L. Li C. (2017). PtoMYB170 positively regulates lignin deposition during wood formation in poplar and confers drought tolerance in transgenic Arabidopsis. *Tree Physiol.* 37 1713–1726. 10.1093/treephys/tpx093 28985414

[B59] Xu R.-F. Li H. Qin R.-Y. Li J. Qiu C.-H. Yang Y.-C. (2015). Generation of inheritable and “transgene clean” targeted genome-modified rice in later generations using the CRISPR/Cas9 system. *Sci. Rep.* 5:11491. 10.1038/srep11491 26089199 PMC5155577

[B60] Xue L.-J. Alabady M. S. Mohebbi M. Tsai C.-J. (2015). Exploiting genome variation to improve next-generation sequencing data analysis and genome editing efficiency in *Populus tremula* x alba 717-1B4. *Tree Genet. Genomics* 11:82 10.1007/s11295-015-0907-5

[B61] Xue L.-J. Tsai C.-J. (2015). AGEseq: analysis of genome editing by sequencing. *Mol. Plant* 8 1428–1430. 10.1016/j.molp.2015.1006.1001 26057235

[B62] Yang L. Zhao X. Ran L. Li C. Fan D. Luo K. (2017). PtoMYB156 is involved in negative regulation of phenylpropanoid metabolism and secondary cell wall biosynthesis during wood formation in poplar. *Sci. Rep.* 7:41209. 10.1038/srep41209 28117379 PMC5259741

[B63] Zhang F. Leblanc C. Irish V. F. Jacob Y. (2017). Rapid and efficient CRISPR/Cas9 gene editing in Citrus using the YAO promoter. *Plant Cell Rep.* 36 1883–1887. 10.1007/s00299-017-2202-4 28864834

[B64] Zhang H. Zhang J. Wei P. Zhang B. Gou F. Feng Z. (2014a). The CRISPR/Cas9 system produces specific and homozygous targeted gene editing in rice in one generation. *Plant Biotechnol. J.* 12 797–807. 10.1111/pbi.12200 24854982

[B65] Zhang Y. Ge X. Yang F. Zhang L. Zheng J. Tan X. (2014b). Comparison of non-canonical PAMs for CRISPR/Cas9-mediated DNA cleavage in human cells. *Sci. Rep.* 4:5405. 10.1038/srep05405 24956376 PMC4066725

[B66] Zhou H. Liu B. Weeks D. P. Spalding M. H. Yang B. (2014). Large chromosomal deletions and heritable small genetic changes induced by CRISPR/Cas9 in rice. *Nucleic Acids Res.* 42 10903–10914. 10.1093/nar/gku806 25200087 PMC4176183

[B67] Zhou X. Jacobs T. B. Xue L.-J. Harding S. A. Tsai C.-J. (2015). Exploiting SNPs for biallelic CRISPR mutations in the outcrossing woody perennial Populus reveals 4-coumarate:CoA ligase specificity and redundancy. *New Phytol.* 208 298–301. 10.1111/nph.13470 25970829

