# Supplementary material for: Genome Editing in Trees: From Multiple Repair Pathways to Long-Term Stability
Source: Front Plant Sci. 2018 Nov 23;9:1732. doi: 10.3389/fpls.2018.01732 (PMC6265510; doi:10.3389/fpls.2018.01732)
Supplement: Supplementary file 1 [file Data_Sheet_1.pdf]

Supplementary Material

Genome Editing in Trees: from multiple repair pathways to long-term stability

William Patrick Bewg, Dong Ci, Chung-Jui Tsai*

*** Correspondence:** Chung-Jui Tsai, cjtsai@uga.edu

1 Supplementary Data

Supplementary Table 1. Repeated amplicon deep-sequencing of previously generated CRISPR mutants.

Supplementary Table S1. Repeated amplicon deep-sequencing of previously generated CRISPR mutants.

Line	Target/ allele	Sequence	Zhou et al. 2015		Repeat analysis	
			%	Pattern	%	Pattern
Potri.001G036900 (4CL1)						
WT	4CL1a	TTGAGGATGATAAAATCTG.GAG**GGG**CTCCATTGGGCAAGGAACTTGAAGATACTGTCAGAGCCAAGTTTC	100%	WT	99%	WT
	4CL1t	TTGAGGATGATAAAATCTG.GAG**GGG**CTCCATTGGGCAAGGAACTTGAAGAAACTGTCAGAGCCAAGTTTC	100%	WT	99%	WT
	4CL5a	TTGAGGATGTTGAAGTCTG.GAGGGTCGCCATTGGGGAAGGAGCTTGAAGATACTGTCAGAGCCAAGTTTC			98%	WT
	4CL5t	TTGAGGATGTTGAAGTCTG.GAGGGTCGCCGTTGGGGAAGGAGCTTGAAGATACTGTCAGAGCCAAGTTTC			99%	WT
Cas9	4CL1a	TTGAGGATGATAAAATCTG.GAGGGGCTCCATTGGGCAAGGAACTTGAAGATACTGTCAGAGCCAAGTTTC	99%	WT	99%	WT
	4CL1t	TTGAGGATGATAAAATCTG.GAGGGGCTCCATTGGGCAAGGAACTTGAAGAAACTGTCAGAGCCAAGTTTC	100%	WT	99%	WT
	4CL5a	TTGAGGATGTTGAAGTCTG.GAGGGTCGCCATTGGGGAAGGAGCTTGAAGATACTGTCAGAGCCAAGTTTC			99%	WT
	4CL5t	TTGAGGATGTTGAAGTCTG.GAGGGTCGCCGTTGGGGAAGGAGCTTGAAGATACTGTCAGAGCCAAGTTTC			99%	WT
4CL1.6	4CL1a	TTGAGGATGATAAAATCT-.GAGGGGCTCCATTGGGCAAGGAACTTGAAGATACTGTCAGAGCCAAGTTTC			100%	-1
	4CL1t	TTGAGGATGATAAAATC-G.GAGGGGCTCCATTGGGCAAGGAACTTGAAGAAACTGTCAGAGCCAAGTTTC	99%	-1	100%	-1
	4CL5a	TTGAGGATGTTGAAGTCTG.GAGGGTCGCCATTGGGGAAGGAGCTTGAAGATACTGTCAGAGCCAAGTTTC			99%	WT
	4CL5t	TTGAGGATGTTGAAGTCTG.GAGGGTCGCCGTTGGGGAAGGAGCTTGAAGATACTGTCAGAGCCAAGTTTC			99%	WT
4CL1.13	4CL1a	TTGAGGATGATAAAATCTGTGAGGGGCTCCATTGGGCAAGGAACTTGAAGATACTGTCAGAGCCAAGTTTC	n.d.		100%	+1
	4CL1t	TTGAGGATGATAAAATCT-.GAGGGGCTCCATTGGGCAAGGAACTTGAAGAAACTGTCAGAGCCAAGTTTC			100%	-1
	4CL5a	TTGAGGATGTTGAAGTCTG.GAGGGTCGCCATTGGGGAAGGAGCTTGAAGATACTGTCAGAGCCAAGTTTC			99%	WT
	4CL5t	TTGAGGATGTTGAAGTCTG.GAGGGTCGCCGTTGGGGAAGGAGCTTGAAGATACTGTCAGAGCCAAGTTTC			99%	WT
4CL1.21	4CL1a	TTGAGGATGATAAAA----.GAGGGGCTCCATTGGGCAAGGAACTTGAAGATACTGTCAGAGCCAAGTTTC	n.d.		100%	-4
	4CL1t	TTGAGGATGATAAAATCT-.GAGGGGCTCCATTGGGCAAGGAACTTGAAGAAACTGTCAGAGCCAAGTTTC			100%	-1
	4CL5a	TTGAGGATGTTGAAGTCTG.GAGGGTCGCCATTGGGGAAGGAGCTTGAAGATACTGTCAGAGCCAAGTTTC			98%	WT
	4CL5t	TTGAGGATGTTGAAGTCTG.GAGGGTCGCCGTTGGGGAAGGAGCTTGAAGATACTGTCAGAGCCAAGTTTC			99%	WT
4CL1.36	4CL1a	TTGAGGATGATAAAATC--.--GGGGCTCCATTGGGCAAGGAACTTGAAGATACTGTCAGAGCCAAGTTTC	n.d.		100%	-4
	4CL1t	TTGAGGATGATAAAATCT-.GAGGGGCTCCATTGGGCAAGGAACTTGAAGAAACTGTCAGAGCCAAGTTTC			100%	-1
	4CL5a	TTGAGGATGTTGAAGTCTG.GAGGGTCGCCATTGGGGAAGGAGCTTGAAGATACTGTCAGAGCCAAGTTTC			99%	WT
	4CL5t	TTGAGGATGTTGAAGTCTG.GAGGGTCGCCGTTGGGGAAGGAGCTTGAAGATACTGTCAGAGCCAAGTTTC			98%	WT
4CL1.37	4CL1a	TTGAGGATGATAAAATCT-.GAGGGGCTCCATTGGGCAAGGAACTTGAAGAAACTGTCAGAGCCAAGTTTC	n.d.		100%	-1
	4CL1t	TTGAGGATGATAAAATCT-.GAGGGGCTCCATTGGGCAAGGAACTTGAAGAAACTGTCAGAGCCAAGTTTC			100%	-1
	4CL5a	TTGAGGATGTTGAAGTCTG.GAGGGTCGCCATTGGGGAAGGAGCTTGAAGATACTGTCAGAGCCAAGTTTC			99%	WT
	4CL5t	TTGAGGATGTTGAAGTCTG.GAGGGTCGCCGTTGGGGAAGGAGCTTGAAGATACTGTCAGAGCCAAGTTTC			99%	WT
4CL1.39	4CL1a	TTGAGGATGATAAAATCT-.GAGGGGCTCCATTGGGCAAGGAACTTGAAGATACTGTCAGAGCCAAGTTTC	100%	-1	100%	-1
	4CL1t	TTGAGGATGATAAAATCT-.GAGGGGCTCCATTGGGCAAGGAACTTGAAGAAACTGTCAGAGCCAAGTTTC	100%	-1	100%	-1
	4CL5a	TTGAGGATGTTGAAGTCTG.GAGGGTCGCCATTGGGGAAGGAGCTTGAAGATACTGTCAGAGCCAAGTTTC			99%	WT
	4CL5t	TTGAGGATGTTGAAGTCTG.GAGGGTCGCCGTTGGGGAAGGAGCTTGAAGATACTGTCAGAGCCAAGTTTC			99%	WT
4CL1.40	4CL1a	TTGAGGATGATAAAATCT-.GAGGGGCTCCATTGGGCAAGGAACTTGAAGATACTGTCAGAGCCAAGTTTC	99%	-1	100%	-1
	4CL1t	TTGAGGATGATAAAATCT-.GAGGGGCTCCATTGGGCAAGGAACTTGAAGAAACTGTCAGAGCCAAGTTTC			100%	-1
	4CL5a	TTGAGGATGTTGAAGTCTG.GAGGGTCGCCATTGGGGAAGGAGCTTGAAGATACTGTCAGAGCCAAGTTTC			99%	WT
	4CL5t	TTGAGGATGTTGAAGTCTG.GAGGGTCGCCGTTGGGGAAGGAGCTTGAAGATACTGTCAGAGCCAAGTTTC			98%	WT
4CL1.45	4CL1a	TTGAGGATGATAAAATCT-.GAGGGGCTCCATTGGGCAAGGAACTTGAAGATACTGTCAGAGCCAAGTTTC	98%	-1	100%	-1
	4CL1t	TTGAGGATGATAAAATCT-.GAGGGGCTCCATTGGGCAAGGAACTTGAAGAAACTGTCAGAGCCAAGTTTC			100%	-1
	4CL5a	TTGAGGATGTTGAAGTCTG.GAGGGTCGCCATTGGGGAAGGAGCTTGAAGATACTGTCAGAGCCAAGTTTC			98%	WT
	4CL5t	TTGAGGATGTTGAAGTCTG.GAGGGTCGCCGTTGGGGAAGGAGCTTGAAGATACTGTCAGAGCCAAGTTTC			98%	WT
4CL1.50	4CL1a	TTGAGGATGATAAAATCTGTGAGGGGCTCCATTGGGCAAGGAACTTGAAGATACTGTCAGAGCCAAGTTTC	100%	+1	100%	+1
	4CL1t	TTGAGGATGATAAAATCT-.GAGGGGCTCCATTGGGCAAGGAACTTGAAGAAACTGTCAGAGCCAAGTTTC			100%	-1
	4CL5a	TTGAGGATGTTGAAGTCTG.GAGGGTCGCCATTGGGGAAGGAGCTTGAAGATACTGTCAGAGCCAAGTTTC			99%	WT
	4CL5t	TTGAGGATGTTGAAGTCTG.GAGGGTCGCCGTTGGGGAAGGAGCTTGAAGATACTGTCAGAGCCAAGTTTC			98%	WT
4CL1.58	4CL1a	TTGAGGATGATAAAATCT-.GAGGGGCTCCATTGGGCAAGGAACTTGAAGATACTGTCAGAGCCAAGTTTC	100%	-1	100%	-1
	4CL1t	TTGAGGATGATAAAATCT-.GAGGGGCTCCATTGGGCAAGGAACTTGAAGAAACTGTCAGAGCCAAGTTTC			100%	-1
	4CL5a	TTGAGGATGTTGAAGTCTG.GAGGGTCGCCATTGGGGAAGGAGCTTGAAGATACTGTCAGAGCCAAGTTTC			100%	WT
	4CL5t	TTGAGGATGTTGAAGTCTG.GAGGGTCGCCGTTGGGGAAGGAGCTTGAAGATACTGTCAGAGCCAAGTTTC			97%	WT
4CL1.60	4CL1a	TTGAGGATGATAAAATCT-.GAGGGGCTCCATTGGGCAAGGAACTTGAAGATACTGTCAGAGCCAAGTTTC	n.d.		100%	-1
	4CL1t	TTGAGGATGATAAAATCT-.GAGGGGCTCCATTGGGCAAGGAACTTGAAGAAACTGTCAGAGCCAAGTTTC			100%	-1
	4CL5a	TTGAGGATGTTGAAGTCTG.GAGGGTCGCCATTGGGGAAGGAGCTTGAAGATACTGTCAGAGCCAAGTTTC			99%	WT
	4CL5t	TTGAGGATGTTGAAGTCTG.GAGGGTCGCCGTTGGGGAAGGAGCTTGAAGATACTGTCAGAGCCAAGTTTC			99%	WT
4CL1.61	4CL1a	TTGAGGATGATAAAATCT-.GAGGGGCTCCATTGGGCAAGGAACTTGAAGATACTGTCAGAGCCAAGTTTC	99%	-1	100%	-1
	4CL1t	TTGAGGATGATAAAATCT-.GAGGGGCTCCATTGGGCAAGGAACTTGAAGAAACTGTCAGAGCCAAGTTTC	99%	-1	100%	-1
	4CL5a	TTGAGGATGTTGAAGTCTG.GAGGGTCGCCATTGGGGAAGGAGCTTGAAGATACTGTCAGAGCCAAGTTTC			99%	WT
	4CL5t	TTGAGGATGTTGAAGTCTG.GAGGGTCGCCGTTGGGGAAGGAGCTTGAAGATACTGTCAGAGCCAAGTTTC			98%	WT
Potri.003G188500 (4CL5)						
WT	4CL5a	GAAAACTTCTCTAAATATCCATCGAAACCTTGTCTGATAAATGGCGCAAAT**GGA**GATATCTACACCTATTC	99%	WT	100%	WT
	4CL5t	GAAAACTTCTCTGAATATCCATCGAAACCTTGTCTGATAAATGGCGAAAAT**GGG**GATATCTACACCTATTC	99%	WT	100%	WT
Cas9	4CL5a	GAAAACTTCTCTAAATATCCATCGAAACCTTGTCTGATAAATGGCGCAAATGGAGATATCTACACCTATTC	99%	WT	100%	WT
	4CL5t	GAAAACTTCTCTGAATATCCATCGAAACCTTGTCTGATAAATGGCGAAAATGGGGATATCTACACCTATTC	98%	WT	100%	WT
4CL5.14	4CL5a	GAAAACTTCTCTAAATATCCATCGAAACCTTGTCTGATAAATGGCGCAAATGGAGATATCTACACCTATTC	99%	WT	99%	WT
	4CL5t	GAAAACTTCTCTGAATATCCATCGAAACCTTGTCTGATAAATGGCGAAAATGGGGATATCTACACCTATTC	99%	WT	100%	WT
4CL5.15	4CL5a	GAAAACTTCTCTAAATATCCATCGAAACCTTGTCTGATAAATGGCGCAAATGGAGATATCTACACCTATTC	99%	WT	99%	WT
	4CL5t	GAAAACTTCTCTGAATATCCATCGAAACCTTGTCTGATAAATGGCGAAAATGGGGATATCTACACCTATTC	99%	WT	100%	WT
4CL5.21	4CL5a	GAAAACTTCTCTAAATATCCATCGAAACCTTGTCTGATAAATGGCGCAAATGGAGATATCTACACCTATTC	100%	WT	99%	WT
	4CL5t	GAAAACTTCTCTGAATATCCATCGAAACCTTGTCTGATAAATGGCGAAAATGGGGATATCTACACCTATTC	99%	WT	99%	WT
4CL5.25	4CL5a	GAAAACTTCTCTAAATATCCATCGAAACCTTGTCTGATAAATGGCGCAAATGGAGATATCTACACCTATTC	100%	WT	99%	WT
	4CL5t	GAAAACTTCTCTGAATATCCATCGAAACCTTGTCTGATAAATGGCGAAAATGGGGATATCTACACCTATTC	99%	WT	100%	WT
4CL5.28	4CL5a	GAAAACTTCTCTAAATATCCATCGAAACCTTGTCTGATAAATGGCGCAAATGGAGATATCTACACCTATTC	99%	WT	100%	WT
	4CL5t	GAAAACTTCTCTGAATATCCATCGAAACCTTGTCTGATAAATGGCGAAAATGGGGATATCTACACCTATTC	100%	WT	100%	WT

The two alleles of *P. tremula* x *alba* sequences (Xue et al., 2015) are shown. PAM sequences are bold underlined and indels in red. Some *4CL1* lines were not sequenced previously (n.d.) or were analyzed in a preliminary experiment using primers that only amplified one allele. Grey sequences in the top panel represent off-target *4CL5* sequences and no cleavage was observed.
